# First report of *Anopheles* (*Cellia*)* multicolor* during a study of tolerance to salinity of *Anopheles arabiensis* larvae in Nouakchott, Mauritania

**DOI:** 10.1186/s13071-020-04400-y

**Published:** 2020-10-16

**Authors:** Mohamed Aly Ould Lemrabott, Gilbert Le Goff, Pierre Kengne, Ousmane Ndiaye, Carlo Costantini, Khadijetou Mint Lekweiry, Mohamed Salem Ould Ahmedou Salem, Vincent Robert, Leonardo Basco, Frédéric Simard, Ali Ould Mohamed Salem Boukhary

**Affiliations:** 1 Laboratoire environnement, santé et société LE2S, Unité de recherche génomes et milieux (jeune équipe associée à l’IRD), Université de Nouakchott Al-Aasriya, BP 880, Nouakchott, Mauritania; 2 MIVEGEC, IRD, CNRS, University of Montpellier, Montpellier, France; 3 CIRMF, Franceville, Gabon; 4 Aix Marseille Univ, IRD, AP-HM, SSA, VITROME, Marseille, France; 5 IHU-Méditerranée Infection, Marseille, France

**Keywords:** *Anopheles multicolor*, *Anopheles arabiensis*, Salinity, Larval ecology, Mauritania

## Abstract

**Background:**

*Anopheles multicolor* is known to be present in the arid areas of Africa north of the Sahara Desert, especially in oases. To date, its presence in Mauritania has not been reported. Here, we present the first record of its presence in Nouakchott, the capital of Mauritania. The larvae of *An. multicolor*, together with those of *An. arabiensis*, the major malaria vector in the city, were found thriving in highly saline surface water collections.

**Methods:**

Entomological surveys were carried out during 2016–2017 in Nouakchott. Mosquito larval habitats were investigated through larval surveys while indoor resting culicid fauna were collected using hand-held aspirator. Physicochemical parameters of the larval habitats were measured on-site, at the time mosquitoes were collected. Larvae and pupae were reared to adults in the insectaries. Morphological and polymerase chain reaction (PCR)-based methods were used to identify newly emerged adults. Batches of fourth-instar larvae were used to assess salinity tolerance by exposing them to increasing concentrations of NaCl, and mortality was monitored throughout development.

**Results:**

Morphological and molecular results confirmed that the specimens were *An. multicolor* and *An. arabiensis*. Sequences of 24 *An. multicolor* adult mosquitoes showed 100% nucleotide identity with the published sequences of *An. multicolor* from Iran. The physicochemical analysis of the water from the two larval habitats revealed highly saline conditions, with NaCl content ranging between 16.8 and 28.9 g/l (i.e. between *c.*50–80% seawater). *Anopheles multicolor* and *An. arabiensis* fourth-instar larvae survival rates at 17.5 g/l NaCl were 86.5% and 75%, respectively. *Anopheles arabiensis* larvae showed variable levels of salt tolerance according to the larval habitat. Adult *An. multicolor* specimens were collected resting indoor at low frequency (0.7%) compared to the other culicid mosquitoes.

**Conclusions:**

To the best of our knowledge, this paper is the first report of *An. multicolor* in Mauritania, extending the known distributional range of the species to the south, as well as to the west. Highly salt-tolerant populations of *An. arabiensis* and *An. multicolor* were observed. Because salt-water collections are widespread in Nouakchott, the relevance of these findings for the dynamics and epidemiology of malaria transmission needs to be assessed.
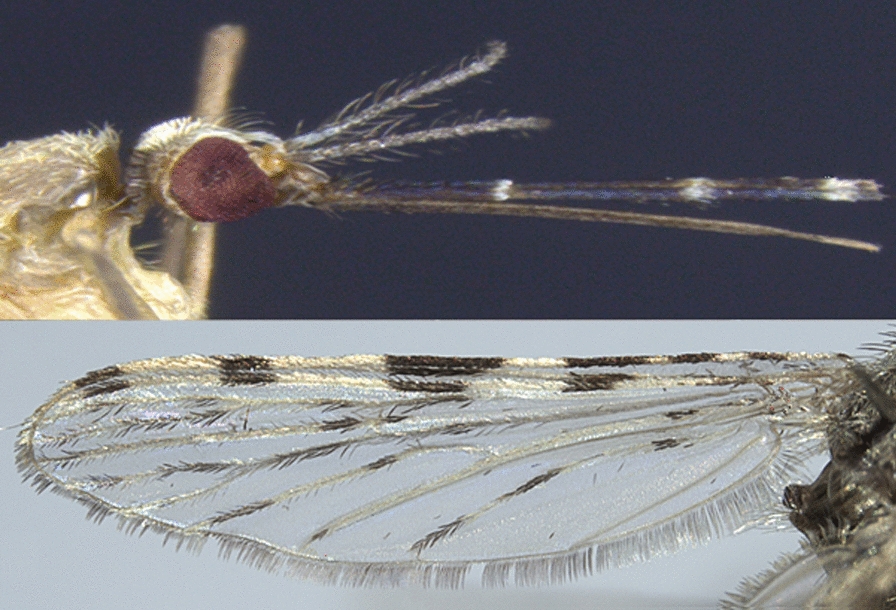

## Background

The genus *Anopheles* (family Culicidae) includes mosquito vectors of human malaria and lymphatic filariasis but is rarely implicated as primary vectors of arboviruses (except for O’nyong-nyong virus). *Anopheles* spp. currently comprise 480 formally recognized species [1], among which 148 are known to occur in the Afrotropical region [2]. Despite their species diversity, fewer than ten are involved in malaria transmission in sub-Saharan Africa and therefore considered of medical importance [3].

Mauritania is a West African country extending from 15°00ʹN to 27°30ʹN and from 05°40ʹW to 17°00ʹW. The country is characterized by two major geoclimatic zones: the Saharan zone to the north and the Sahelian zone to the south. These two zones present a marked difference in climatic and geographic conditions. Nouakchott, the capital city of Mauritania, is situated in the Saharan zone. Malaria has become endemic in Nouakchott over the past two decades, with *Plasmodium vivax* being the predominant causative parasite transmitted by *Anopheles arabiensis*, the major vector [4–7]. The principal factors that most likely explain the emergence followed by endemicity of malaria in Nouakchott were reviewed [6]. The peak malaria transmission occurs in October and November, i.e. a few weeks after the rainy season, although malaria cases, mostly due to *P. vivax*, have been reported throughout the year [5].

A comprehensive literature review conducted in 2017 reported that 17 anopheline species have so far been described in Mauritania, including three members of the *An. gambiae* complex: *An. arabiensis*, *An. gambiae* (*s.s.*) and *An. melas* [8]. *Anopheles arabiensis* is widely distributed in the Sahelian as well as the Saharan zone of the country [7, 9, 10]. Moreover, previous studies have shown that it is the only malaria vector encountered in Nouakchott [11, 12]. In this urban area, *An. arabiensis* larvae were found in a variety of freshwater bodies, such as water discharged from standpipes and household drinking water tanks [11, 12].

*Anopheles* (*Cellia*) *multicolor* Cambouliu, 1902 has been reported from arid areas in Africa north of the Sahara Desert, from Morocco to Egypt, extending further south in the African continent along the Nile river valley and the Red Sea coast [13–18]. *Anopheles multicolor* was also reported in the Asian continent, particularly in India, Pakistan and Iran [16, 19]. In sub-Saharan Africa, its presence was reported only in Niger at the extreme northern limit of the country [20, 21]. The larvae of *An. multicolor* can be found in freshwater collections and cesspools, but they usually develop in brackish water pools and may also grow in highly saline water (up to 50–65% seawater) in coastal and inland areas [15, 22–25]. They also develop in polluted water collections in urban areas [14, 20, 24]. Although there is no report of *An. multicolor* mosquitoes naturally infected with *Plasmodium* spp., its role in malaria transmission has long been suspected, especially in some Saharan oases where female adult anophelines were captured during malaria epidemics [13, 14, 24, 26].

During October 2016, routine vector surveillance was carried out in various mosquito larval habitats in Nouakchott as part of entomological studies to assess salinity tolerance of *An. gambiae* (*s.l.*). The first larval samples collected in two salty larval habitats in the vicinity of the former Nouakchott international airport and raised in insectaries emerged as morphologically distinct adult anopheline specimens, together with *An. arabiensis* as the single member of the *An. gambiae* complex in that area. These morphological observations led to the demonstration that a second species of *Anopheles* was present in Nouakchott. The present study provides the first record of *An. multicolor* in Mauritania and evidence that *An. multicolor* and *An. arabiensis* co-exist at larval stage in the same breeding places in Nouakchott and are capable of developing in highly saline water.

## Methods

### Study site

**Fig. 1:**
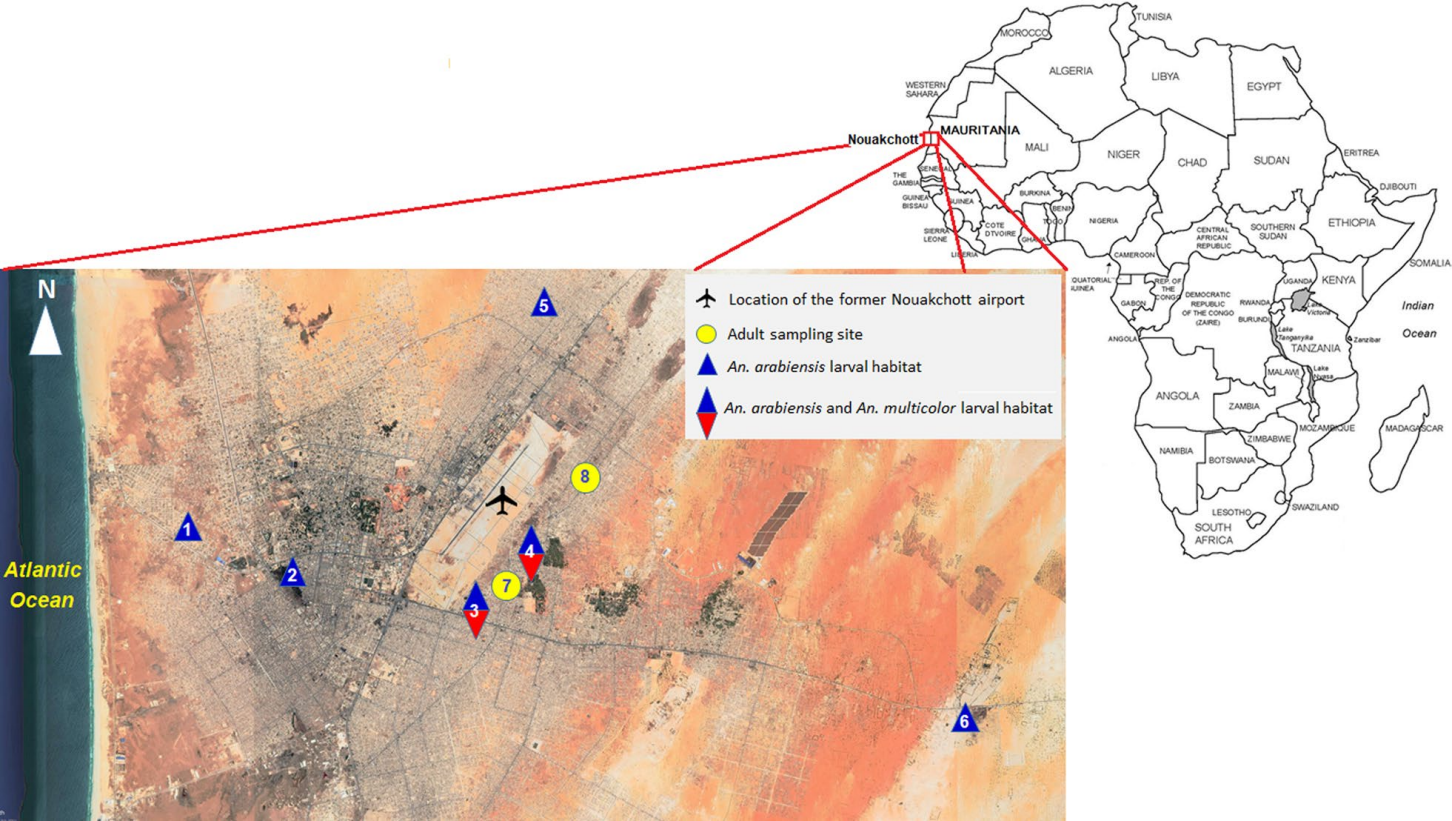
Distribution of the mosquito larvae and adults sampling sites in Nouakchott, Mauritania. Larval sampling sites: 1, Cité plage; 2, Sebkha; 3, Ten Soueilim; 4, Zaatar; 5, Ain Talh; 6, Tenweish. Adult sampling sites: 7, Ten Soueilim; 8, Zaatar

The present study was conducted in Nouakchott (18°05ʹN, 15°58ʹW) between October 2016 and November 2017. Nouakchott is located along the Atlantic coast in the Saharan zone of Mauritania, a few meters below (− 3 m) or above (up to 10 m) sea level (Fig. 1). The capital city is comprised of nine districts, with a total of nearly 1,000,000 inhabitants. Its population density is 800 inhabitants per km^2^. The city center is situated about 5 km from the Atlantic coast. A 1–2 km wide salty depression, locally known as “Sebkha” separates the city confines from the sea. Currently, 27% of the inhabitants of Nouakchott are living in this area [27].

The climate in Nouakchott is characterized by a long dry season (October-June) and a short rainy season (July-September). The mean annual rainfall during the period 1970–2013 in Nouakchott was 89.5 mm [28] with most of the rainfall occurring between August and mid-September. The average annual minimum and maximum temperatures in Nouakchott are 20.5 °C (range 18.5–22.5 °C) and 33°C (range 31.4–34.5 °C), respectively [28]. The annual relative humidity ranges from 33% to 79%. Wind is mainly bidirectional and very erosive, with winds blowing from the Northeast from October to May, and from the north-west from June to September (National Meteorological Office, unpublished data; https://fr.meteo-mauritanie.org/).

Nouakchott is facing many environmental challenges. One of the most notable urban problems includes the rise of the water table, resulting in large areas of brackish water pools seeping through the soil and disseminating across the city, particularly in areas below sea level. The city also suffers from recurrent seasonal floods after ‘heavy’ rains due to the lack of effective water evacuation system, canalization, and sanitation.

### Physicochemical characteristics of anopheline larval habitats

A total of six natural and man-made anopheline aquatic habitats located within a distance of 1–18 km from the Atlantic coast in the districts of Cité plage, Sebkha, Ten Soueilim, Zaatar, Ain Talh, and Tenweish were prospected monthly from October 2016 to June 2017, during the dry season (Fig. 1). Physicochemical parameters of the larval habitats were measured on-site between 12:00 and 14:00. Salinity (g/l), dissolved oxygen (%), pH, water temperature (°C), and turbidity (nephelometric turbidity units, NTU) were measured using a multiparameter apparatus (HI 98194 and HI 93703, Hannah, Nusfalau, Romania). Other characteristics of the larval habitats, such as size, depth, presence/absence of vegetation and light exposure, were also recorded.

### Collection and morphological identification of mosquitoes

Anopheline immature stages were collected in October 2016, January 2017 and May 2017 using 350 ml standard mosquito dippers (BioQuip Products, Rancho Dominguez, CA, USA) from the six prospected larval habitats, and transported to insectaries at the University of Nouakchott Al-Aasriya, Nouakchott, Mauritania. Larvae and pupae were reared to adult stage in their field-collected water, and set in rearing cages under a photoperiod of 12 h light:12 h dark, ambient temperature of 28 ± 2 °C, and a humidity of 75 ± 5%. Emerging adult mosquitoes were collected from the rearing cages using a mouth aspirator (John W. Hock Co., Gainesville, FL, USA), and frozen at – 20 °C for 30 min before morphological identification. Batches of fourth-instar larvae (L4) were also used for the salinity tolerance assays.

In parallel, collection of indoor resting culicid fauna was undertaken from November 2016 to October 2017 using a battery powered Prokopack aspirator (John W. Hock Co.). The collection was carried out once monthly during the morning (between 07:00 to 09:00) in two sites close to the larval habitats where *An. multicolor* specimens were found. Oral consent was obtained from each household head prior to indoor mosquito collection. Captured mosquitoes were frozen and then sorted on chill table into their respective genus, *Anopheles*, *Culex* or *Aedes*.

Fourth-instar larvae and adult females were morphologically identified using standard taxonomic keys and softwares [23, 29–33]. The specimens were examined and photographed under a Leica Z16 APO A stereomicroscope (Leica Microsystems, Wetzlar, Germany). Mosquito species names were assigned according to Harbach [1]. All morphologically identified mosquitoes belonging to the genus *Anopheles* were further analyzed by polymerase chain reaction (PCR) to confirm morphological identification.

### Salinity tolerance of anopheline larvae

Batches of 15 fourth-instar larvae of *Anopheles* mosquitoes collected from each larval habitat were exposed to different saline concentrations obtained by diluting seawater with distilled water assuming that 100% seawater is equivalent of 35 g/l of NaCl [34, 35]. These were 3.5 g/l NaCl (10% seawater), 7.0 g/l NaCl (20% seawater), 10.5 g/l NaCl (30% seawater), 14 g/l NaCl (40% seawter) and 17.5 g/l NaCl (50% seawater). Distilled water was used as control (i.e. 0% seawater). Larvae were placed in 150 ml plastic cups containing 100 ml of the different saline concentrations and incubated at room temperature for 24 h. The cups were covered with plastic lids to reduce evaporation. Two replicate tests were run in parallel for each NaCl concentration. At the end of exposure interval, the number of surviving larvae at each salinity level was counted, and the results were recorded as the mean percentage of surviving larvae for each salt concentration. Dead larvae at each salt concentration were removed, and those that survived the experimental growth conditions were allowed to emerge into adults for morphological and molecular identification.

### Molecular identification of *Anopheles* mosquito species

Molecular identification of anopheline mosquitoes was performed on adult mosquitoes. Genomic DNA was extracted from the legs and wings of a single female mosquito with 2% cetyltrimethylammonium bromide (CTAB) as described by Morlais et al. [36]. Dried DNA pellets were re-suspended in 20 μl of 1× Tris-ethylenediaminetetraacetic acid (EDTA) buffer and stored at – 20 °C. DNA samples of the specimens identified morphologically as *An. gambiae* (*s.l.*) were used as template in the PCR method to differentiate sibling species of *An*. *gambiae* complex [37]. For specimens morphologically identified as *An. multicolor*, the ribosomal DNA (rDNA) fragment located between the 3ʹ-end of the *5.8S* subunit and the 5ʹ-end of *28S* subunit was amplified by PCR [38, 39]. PCR products were sequenced using ABI 3130XL apparatus (Applied Biosystems, Villebon-sur-Yvette, France). Sequences were edited using Genetics Computer Group (GCG) genetic sequence analysis software package (“Wisconsin package”) (GCG, Madison, WI, USA) [40] and compared with *An. multicolor* ribosomal RNA gene sequence deposited in GenBank (GenBank: AY564228) using MEGA4.0 software [41].

### Statistical analysis

Data were analyzed using MedCalc software for Windows, version 15.0 (MedCalc Software, Ostend, Belgium). The mean ± standard deviation (SD) percentage of surviving larvae was calculated for each saline concentration using the following formula:

Percent surviving larvae = (No. of surviving larvae/No. of total larvae exposed to the same NaCl concentration) × 100

Proportions were compared using the Chi-square test. A *P-*value < 0.05 was considered statistically significant.

## Results

### Morphological features

The larvae and adult stages of *An. multicolor* mosquitoes collected in Nouakchott possessed all the species-specific morphological characters reported in the identification keys.

The larvae of *An. multicolor* have simple, smooth clypeal bristles (2-C and 3-C) and weakly developed shoulder bristles (1-P and 2-P). As a member of the *An*. (*Cellia*) *listeri*-group, *An. multicolor* has one simple meso-pleural bristle (9-M and 10-M), another aciculate, and two branchy meta-pleural bristles (9-T and 10-T). The palmate setae are weakly developed and generally have simple leaves with no terminal filament. The palmate setae are developed only from the third abdominal tergite (setae 1-III).

**Fig. 2:**
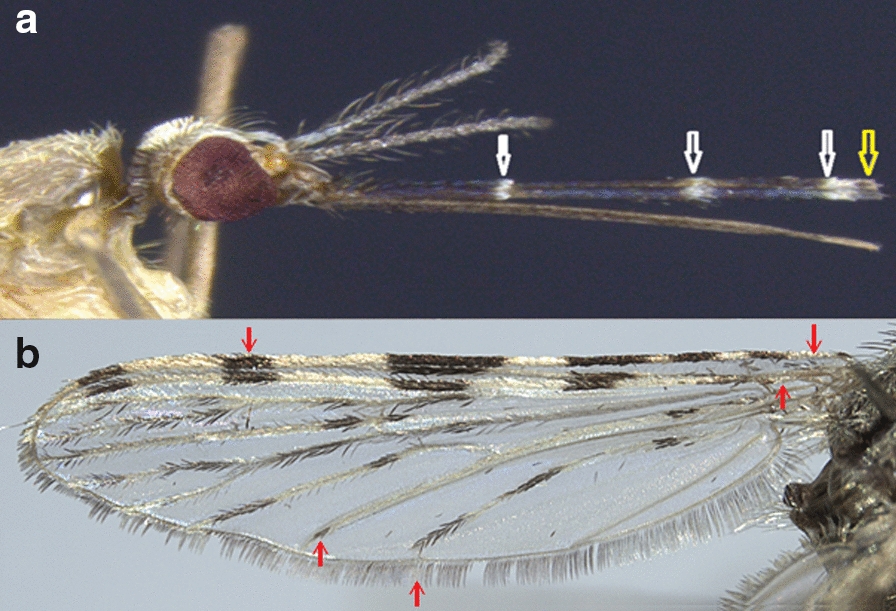
Head and wing of *Anopheles multicolor* female mosquito captured in Nouakchott, Mauritania. White and yellow arrows in (**a**, head) show the clear rings and dark terminal ring that characterize *An. multicolor* maxillary palps, respectively. Arrows in (**b**, wing) show (i) the sectorial clear zone and the median dark area on the costa, and (ii) the dark apical spot on the posterior cubital vein of the wing of *An. multicolor* adult females

Adult females have maxillary palps with 3 clear rings (Fig. 2a) and a dark terminal ring. The wings are largely pale (Fig. 2b). The following morphological criteria of the wings are particularly relevant to identify *An*. *multicolor*: (i) wide clear spot at the base of the wing (costa sub-costa and radial); (ii) wide clear spots on the costal veins, subcostal and radial R1 involving in particular the reduced width of the preapical black spot; and (iii) on the wing fringe, absence of a clear spot opposite the anal vein. The legs are generally dark with very small clear apical spots on the shins, which do not form complete rings (not shown).

### Molecular identification

For further characterization of *An. multicolor* specimens, a single band of 688 bp in the internal transcribed spacer 2 (ITS2) of the rRNA gene cluster was amplified from 24 adult females. Nucleotide sequences obtained from each of these samples revealed the same sequence (GenBank accession number MT790357) in all tested specimens. Analysis with the Basic Local Alignment Search Tool (BLAST) revealed the best fit with the published sequences of *An. multicolor* from Iran (GenBank: AY564228). The global alignment and comparison of all 24 sequences from adult females with that of *An. multicolor* showed no genetic variability (100% nucleotide similarity).

### Characterization of larval habitats

Among prospected surface water collections, only 6 were positive for mosquito immature stages, including 2 natural and 4 man-made larval habitats (Table 1). Natural larval habitats consisted of 1 puddle (Ten Soueilim district) and 1 pool (Zaatar district) of salty ground-water, while man-made habitats included 3 puddles; of which one from Ain Talh district was formed by water discharged from a public stand pipe and two from Cité plage and Tenweish districts originated from decrepit water pipes. The sixth larval habitat (Sebkha district) was a pit in a vegetable garden.

**Table 1:** Characteristics of larval habitats of *Anopheles multicolor* and *An. arabiensis* in Nouakchott, Mauritania

District	GPS (North, West)	Altitude (m)	Habitat type	Origin	Salinity (g/l)	Turbidity (NTU)	D.O (%)	*Anopheles* spp. larvae	
								*An. arabiensis*	*An. multicolor*
Ain Talh	18°07ʹ36ʺ, 15°56'11ʺ	7	Puddle	Public standpipe	0.2	44.7	50.7	+	−
Tenweish	18°03ʹ23ʺ, 15°51ʹ00ʺ	5	Puddle	Drinking water pipe	0.6	9.8	55.0	+	−
Sebkha	18°05ʹ02ʺ, 15°59ʹ17ʺ	7	Pit	Market gardening	1.3	101.0	28.2	+	−
Cité plage	18°05ʹ57ʺ, 16°00ʹ09ʺ	3	Puddle	Drinking water pipe	2.3	199.4	24.6	+	−
Ten Soueilim	18°05ʹ57ʺ, 15°57ʹ09ʺ	4	Pool	Ground-water	16.8	10.0	65.0	+	+
Zaatar	18°05ʹ37ʺ, 15°56ʹ20ʺ	5	Puddle	Ground-water	28.9	35.4	29.7	+	+

*Abbreviations*: GPS, global positioning system; NTU, Nephelometric turbidity unit; D.O, dissolved oxygen; m, meters above sea level

The size of the larval habitats did not exceed 2 m^2^ with a maximum depth of less than 20 cm. They were all well exposed to the sun and had no vegetation, except for the larval habitat in Ten Soueilim district, which was formed from ground-water with the presence of *Typha* spp., a perennial herbaceous plant. The mean (± SD) water temperature was 28.8 ± 2.7 °C, with a range of 24.8–32.2 °C. Salt contents were lowest in man-made larval habitats (0.2–2.3 g/l NaCl) and highest in natural larval habitats situated in Ten Soueilim and Zaatar districts, with a salt content of 16.8 and 28.9 g/l NaCl, respectively. The turbidity of larval habitats was low (9.8–10 NTU) to medium (19.6–101.0 NTU), and only the larval habitat situated in Cité plage was classified as turbid (199.4 NTU). Dissolved oxygen in the larval habitats varied between 24.6% in Cité plage and 65% in Ten Soueilim, with a mean (± SD) of 40 ± 16.1%. The six larval habitats contained larvae of *An. arabiensis* while *An. multicolor* and *An. arabiensis* larvae were found together in Ten Soueilim and Zaatar natural larval habitats characterized by the highest salt concentration measured in this study (Table 1).

### Indoor resting mosquitoes

A total of 1600 indoor resting mosquitoes were collected, including 415 (26%) *Aedes aegypti*, 804 (50.2%) *Culex quinquefasciatus*, 261 (16.3%) *Cx. decens*, 45 (2.8%) *Cx. tritaeniorhynchus*, 23 (1.4%) *Cx. univittatus*, 41 (2.5%) *Anopheles arabiensis*, and 11 (0.7%) *An. multicolor* (Table 2). Of 11 female *An. multicolor* captured, 7 were unfed, 4 were blood-fed, and none were gravid or semi-gravid. All 11 female *An. multicolor* mosquitoes were captured during the dry season of 2017.

**Table 2:** *Anopheles multicolor* and other indoor-resting *Culicidae* collected using hand-held aspirator in Nouakchott between March and November 2017

Genus	Species	*n* (%)
*Aedes*	*Ae.* (*Stegomyia*) *aegypti*	415 (26)
*Anopheles*	*An.* (*Cellia*) *arabiensis*	41 (2.5)
	*An.* (*Cellia*) *multicolor*	11 (0.7)
*Culex*	*Cx.* (*Culex*) *quinquefasciatus*	804 (50.2)
	*Cx.* (*Culex*) *decens*	261 (16.3)
	*Cx.* (*Culex*) *tritaeniorhynchus*	45 (2.8)
	*Cx.* (*Culex*) *univittatus*	23 (1.4)
Total		1600 (100)

*Abbreviation*: n, number of mosquitoes

### Salinity tolerance

**Fig. 3:**
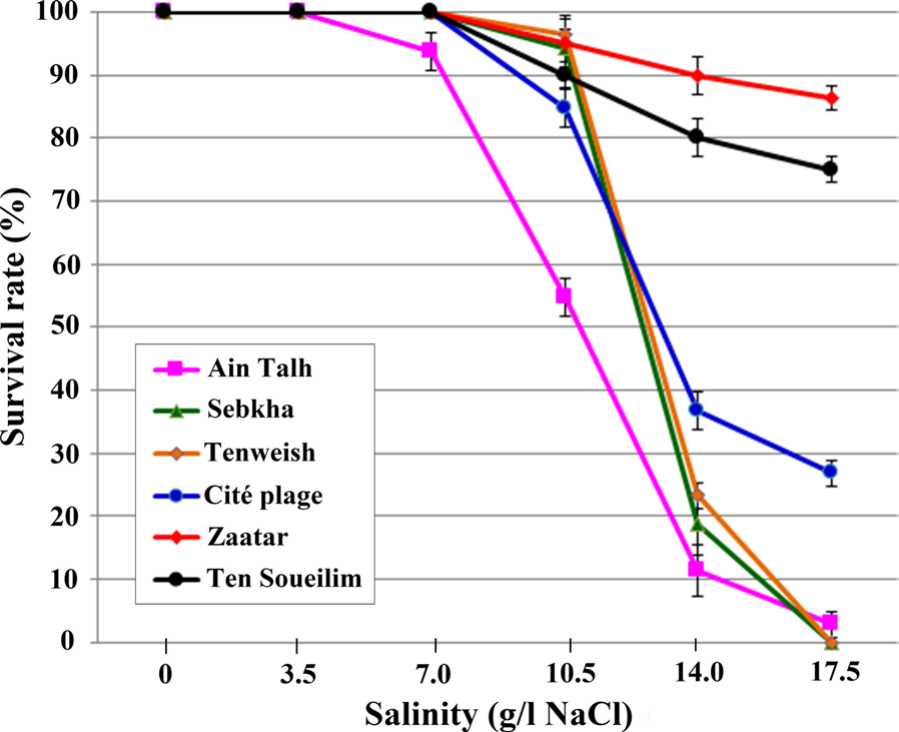
Salinity tolerance test with fourth-instar larvae collected from six larval habitats in Nouakchott, Mauritania. A total of 180 fourth-instar larvae from each of the six larval habitats were exposed to increasing salt concentration from 0 to 17.5 g/l NaCl for 24 h

A total of 180 fourth-instar anopheline larvae from each larval habitat were exposed to an increasing salt concentration from 0 (100% distilled water) to 17.5 g/l NaCl for 24 h. Results of salinity test are summarized in Fig. 3.

Larval survival was 100% in distilled water regardless of the origin of their larval habitats. For freshwater larval habitats of Ain Talh, Sebkha, Cité plage and Tenweish, where salinity was less than 2.3 g/l, larval survival was 100% at 3.5 g/l NaCl. For larvae sampled from Ain Talh, 100% survival was observed at 3.5 g/l NaCl, while 100% survival was obtained in 7.0 g/l NaCl for those collected from Sebkha, Cité plage and Tenweish larval habitats. The survival rate began to decline at 7.0 g/l NaCl for larvae sampled from Ain Talh and at 10.5 g/l NaCl for those of the remaining three larval habitats.

While none of the sampled larvae from Sebkha and Tenweish survived at 17.5 g/l NaCl, there were still 26.8% (*n *= 8) and 2.8% (*n *= 1) larvae alive among those from Cité plage and Ain Talh larval habitats, respectively (*χ*^2^ = 0.249, *df *= 1, *P *= 0.61). All emerged adult mosquitoes from the four larval habitats (*n *= 498) belonging to the *An. gambiae* species complex were identified as *An. arabiensis* by PCR*.*

Higher survival rates were observed among larvae collected from the two saltwater larval habitats of Zaatar and Ten Soueilim where larvae showed 100% survival at salinity levels of 0 g/l NaCl (i.e. distilled water), 3.5 g/l NaCl and 7.0 g/l NaCl. Survival of anopheline larvae from Zaatar decreased to 86.5% after 24 h exposure to 17.5 g/l NaCl. In comparison, the survival rate of larvae from Ten Soueilim exposed to 17.5 g/l NaCl was 75%. The difference in the survival rates of larvae collected at these two larval habitats was not significant (*χ*^2^ = 0.973, *df* = 1, *P* = 0.32). Surviving larvae (*n* = 335) from the two larval habitats were reared to adult stage. One hundred eighty-five (55.2%) mosquitoes were morphologically identified as *An. multicolor* and 150 (44.8%) as *An. arabiensis.*

## Discussion

The results of the present study establish, to our knowledge for the first time, the presence of breeding specimens of *An. multicolor*, a salt-tolerant anopheline species, in Nouakchott. This is the westernmost record of *An. multicolor* so far and the southernmost record in continental Africa and even in the world [18, 23]. The presence of *An. multicolor* was confirmed using both morphological criteria and molecular evidence based on ITS sequences.

*Anopheles multicolor* was known only from the arid areas of northern Africa, particularly in oases and in the Nile Delta. Its larvae were found thriving in habitats with high levels of salinity, i.e. up to 14-35 g/l NaCl (40–100% seawater) [17]. Despite its wide geographical distribution, the biology of this species is still poorly known. *Anopheles multicolor* bites both humans and animals indoors and outdoors. A marked preference for animal blood (up to 85%) has been reported in Algeria and Egypt [13, 42]. Females also enter into houses and seek for humans for their blood meal. Hammadi et al. [15] reported a marked anthropophilic rate of 64% in the region of Tamanrasset in southern Algeria. In the present study which lasted for 12 months, only 11 indoor resting female *An. multicolor* were collected, including 7 unfed, 4 blood-fed, and 0 gravid or semi-gravid, suggesting a low preference for resting in human shelters and a marked tendency of female mosquitoes to complete part, if not all of their gonotrophic cycles, outside dwellings. Similar findings were reported from Egypt [13].

*Anopheles multicolor* has never been found with *Plasmodium* sporozoites in natural settings, by either dissection of salivary glands or enzyme-linked immunosorbent assay (ELISA) or PCR tests, although it has been successfully infected with *P. falciparum* in experimental conditions in the insectaries [34]. Nevertheless, *An. multicolor* may be involved in malaria transmission [13, 15, 43–47]. The origin of *An. multicolor* mosquitoes in Nouakchott is still unknown. Its presence was reported in Morocco and Algeria, two countries bordering Mauritania. Hence, *An. multicolor* may have been introduced from these countries through passive dispersal or human-assisted adult and/or egg transportation by car or airplane. A possible introduction of adult mosquitoes by airplane cannot be excluded because the larval habitats where *An. multicolor* larvae were found are located in the vicinity of the former international airport of Nouakchott. Further population genetic and genomic studies should help to disentangle these hypotheses.

An important finding of the present study is the presence of larvae of *An. arabiensis* developing together with those of *An. multicolor* in highly saline surface water in Nouakchott. *Anopheles arabiensis* is a member of the *An. gambiae* (*s.l.*) species complex which includes the main malaria vectors in Africa. Currently, *An. gambiae* complex comprises nine recognized sibling species, of which only *An. melas*, *An. merus* and *An. bwambae* are saltwater tolerant, with larvae reported to survive in larval habitats with high saline levels reaching up to 64.7 and 70.4 g/l NaCl for *An. merus* and *An. melas*, respectively [23, 48]. Therefore, these two species are commonly associated with coastal malaria transmission [49, 50]. The other sibling species in the complex, *An. gambiae* (*s.s.*), *An. coluzzii*, *An. arabiensis*, and *An. fontenillei*, are obligate freshwater species, and 10–12 g/l NaCl (about 30% seawater) is considered the upper salinity limit for normal larval development of *An. gambiae* and *An. coluzzii* [51–53]. For *An. arabiensis*, studies testing salinity tolerances are scarce. A report dating back to 1947 showed that aquatic stages of *An. arabiensis* could tolerate chloride levels of 4.44 g/l [54]. In the present study, the high salinity tolerance of *An. multicolor* and *An. arabiensis* larvae observed during laboratory experiments (75% and 86.5% larval survival after exposure to 17.5 g/l NaCl for 24 h) corroborates their presence in highly brackish larval habitats from which they were sampled in Nouakchott. This level of salt tolerance is comparable to that reported for saltwater sibling species of *An. gambiae* (*s.l.*) [23, 45, 49]. The study also revealed the presence of larvae of *An. arabiensis* in freshwater habitats in Nouakchott, such as those of Ain Talh, Tenweish, and Sebkha, suggesting the presence of locally adapted ecotypes to variable larval habitat conditions. This observation is consistent with the results of the studies that reported that saltwater tolerant mosquito larvae can develop normally in both fresh and saline habitats [52]. The presence of both fresh and brackish larval habitats in Nouakchott is the result of artificial (man-made) and natural (rainfall and rising goundwater) factors [11, 12]. During the past decade, a succession of dry years (2012, 2015 and 2017) and humid years, with sometimes heavy rainfall (floods in 2013 and 2016), occurred in Nouakchott (National Meteorological Office, unpublished data). This climatic situation resulted in the presence of numerous temporary and semi-permanent larval habitats with different and fluctuating levels of salinity, depending on their altitude and the amount of rainfall. In a year marked by low rainfall, water evaporates in the majority of freshwater habitats during the long dry season (October-June), which results in increased salinity in most surface water collections, thus exerting a selection pressure on mosquito larvae. Hence, the presence of salt-tolerant *An. arabiensis* could be considered as an adaptive trait that allows larvae to complete aquatic stages and maintain mosquito species in the arid conditions of Nouakchott. Furthermore, brackish or saline sites were reported to contain high nutrient levels coupled with reduced competition and predation, particularly in those sites subjected to rapid changes in salinity [55].

Salt-tolerant anopheline mosquito species are commonly thought to be poor malaria vectors compared to freshwater species [36]. In the present study, the observed salt tolerance of malaria vectors, particularly *An. arabiensis*, could be of relevance for malaria epidemiology in the city.

The effect of salinity on morphological characteristics, such as the length of anal papillae in *An. multicolor* and *An. arabiensis*, and the physiological basis that governs salinity tolerance in these two species were not investigated here but should be part of future studies. Indeed, Coetzee & Le Sueur [56] noted that anal papillae of *An. merus* larvae raised in freshwater was twice as long as those in larvae reared in saline water allowing osmoregulation within the larva. Furthermore, urbanization leads to the exposure of members of *Anopheles* vectors to a range of water pollution in urban settings. Some studies documented an obvious tolerance of *An. arabiensis* to urban polluted larval habitats accompanied by resistance to larvicide [57], which would be interesting to combine with studies on the salinity tolerance of *Anopheles* larvae.

The present study also showed that *An. arabiensis* larvae can thrive in small- to medium-sized aquatic habitats fully exposed to the sun with both clear and turbid water, little vegetation and within a wide range of dissolved oxygen. *Anopheles multicolor* larvae also thrive in aquatic habitats with similar characteristics with the exception that water was less turbid. Similar results were previously reported for *An. arabiensis* [11, 12] and *An. multicolor* [17].

## Conclusions

The results of the present study showed one of the highest levels of salinity documented so far in a larval habitat for the freshwater malaria vector, *An. arabiensis*, and extend the known distribution range of *An. multicolor* to the South and to the West on the African continent. Because salt-water bodies are widespread in Nouakchott, the relevance of these findings for the dynamics and epidemiology of malaria transmission needs to be further investigated. Moreover, further studies are required to demonstrate the vectorial capacity of *An. multicolor*, in particular in Nouakchott, and to determine its potential role in malaria transmission.

## Data Availability

All datasets generated or analyzed during the present study are presented within the article.

## Abbreviations

BLAST: Basic Local Alignment Search Tool
CTAB: Cetyltrimethylammonium bromide
EDTA: Ethylenediaminetetraacetic acid
ELISA: Enzyme-linked immunosorbent assay
GPS: Global positioning system
ITS2: Internal transcribed spacer 2
NaCl: Sodium chloride
NTU: Nephelometric turbidity units
PCR: Polymerase chain reaction
rDNA: Ribosomal DNA
rRNA: Ribosomal RNA
SD: Standard deviation

## References

[1] Harbach RE. Mosquito taxonomic inventory, 2013. https://mosquito-taxonomic-inventory.info/. Accessed 01 July 2020.

[2] Irish SR, Kyalo D, Snow RW, Coetzee M (2020). Updated list of *Anopheles* species (Diptera: Culicidae) by country in the Afrotropical region and associated islands. Zootaxa.

[3] Manguin S, Carnevale P, Mouchet J, Coosemans M, Julvez J, Richard-Lenoble D, Sircoulon J (2008). Biodiversity of malaria in the world.

[4] Cortes H, Morillas-Marquez F, Valero A (2003). Malaria in Mauritania: the first cases of malaria endemic to Nouakchott. Trop Med Int Health..

[5] Mint Lekweiry K, Basco LK, Ould Ahmedou Salem MS, Hafid JE, Marin-Jauffre A, Ould Weddih A (2011). Malaria prevalence and morbidity among children reporting at health facilities in Nouakchott, Mauritania. Trans R Soc Trop Med Hyg.

[6] Mint Lekweiry K, Ould Ahmedou Salem MS, Basco LK, Briolant S, Hafid J, Ould Mohamed Salem Boukhary A (2015). Malaria in Mauritania: retrospective and prospective overview. Malar J.

[7] Mint Lekweiry K, Ould Ahmedou Salem MS, Cotteaux-Lautard C, Jarjaval F, Marin-Jauffre A, Bogreau H (2016). Circumsporozoite protein rates, blood-feeding pattern and frequency of knockdown resistance mutations in *Anopheles* spp. in two ecological zones of Mauritania. Parasit Vectors.

[8] Mint Mohamed Lemine A, Ould Lemrabott MA, Ebou MH, Mint Lekweiry K, Ould Ahmedou Salem MS, Ould Brahim K (2017). Mosquitoes (Diptera: Culicidae) in Mauritania: a review of their biodiversity, distribution and medical importance. Parasit Vectors.

[9] Dia I, Ba H, Ould Mohamed SA, Diallo D, Lo B, Diallo M (2009). Distribution, host preference and infection rates of malaria vectors in Mauritania. Parasit Vectors.

[10] Ould Lemrabott MA, Ould Ahmedou Salem MS, Ould Brahim K, Brengues C, Rossignol M, Bogreau H (2018). Seasonal abundance, blood meal sources and insecticide susceptibility in major anopheline malaria vectors from southern Mauritania. Parasit Vectors.

[11] Ould Ahmedou Salem MS, Mint Lekweiry K, Mint Hasni M, Konate L, Briolant S, Faye O (2013). Characterization of anopheline (Diptera: Culicidae) larval habitats in Nouakchott, Mauritania. J Vector Borne Dis.

[12] Mint Lekweiry K, Ould Ahmedou Salem MS, Ould Brahim K, Ould Lemrabott MA, Brengues C, Faye O (2015). *Aedes aegypti* in Mauritania: first report on the presence of the arbovirus mosquito vector in Nouakchott. J Med Entomol.

[13] Kenawy MA, Beier JC, El Said S (1986). First record of malaria and associated *Anopheles* in El Gara oasis Egypt. J Am Mosq Control Assoc.

[14] Ramsdale CD (1990). *Anopheles* mosquitoes and imported malaria in Libya. Mosquito Syst.

[15] Hammadi D, Boubidi SC, Chaib SE, Saber A, Khechache Y, Gasmi M (2009). Le paludisme au Sahara algérien. Bull Soc Pathol Exot.

[16] Harbach RE, Manguin S (2013). The phylogeny and classification of *Anopheles*. Anopheles mosquitoes—new insights into malaria vectors.

[17] Tabbabi A, Boussès P, Rhim A, Brengues C, Daaboub J, Ben-Alaya-Bouafif N (2015). Larval habitats characterization and species composition of *Anopheles* mosquitoes in Tunisia, with particular attention to *Anopheles maculipennis* complex. Am J Trop Med Hyg.

[18] Trari B, Dakki M, Harbach RE (2017). An updated checklist of the Culicidae (Diptera) of Morocco, with notes on species of historical and current medical importance. J Vector Ecol.

[19] Sedaghat MM, Harbach RE (2005). An annotated checklist of the *Anopheles* mosquitoes (Diptera: Culicidae) in Iran. J Vector Ecol.

[20] Julvez J, Mouchet J, Suzzoni J, Larrouy G, Fouta A, Fontenille D (1998). Les anophèles du Niger. Bull Soc Pathol Exot.

[21] Coetzee M (2020). Key to the females of Afrotropical *Anopheles* mosquitoes (Diptera: Culicidae). Malar J.

[22] Buxton PA (1944). Rough notes: *Anopheles* mosquitoes and malaria in Arabia. Trans R Soc Trop Med Hyg.

[23] Gillies MT, De Meillon B (1968). The Anophelinae of Africa south of the Sahara (Ethiopian zoogeographical region). Publ South Afr Inst Med Res.

[24] Chauvet G, Hassan NT, Izri MA (1985). Problèmes palustres et route transsaharienne. Bull Soc Pathol Exot.

[25] Trari B, Fontenille D, Agoumi A (2004). Le paludisme à Larache: recherche de l’infection plasmodiale chez les anophèles (Diptera: Culicidae) par une technique immuno-enzymatique. Intérêt de l’utilisation de la technique au Maroc. Animalis.

[26] Holstein M, Le Corroller Y, Addadi K, Guy Y (1970). The *Anopheles* of the Sahara. Arch Inst Pasteur Alger.

[27] Office National des Statistiques. Recensement Général de la Population et de l’Habitat (RGPH) 2013. Chapitre 1. Répartition spatiale de la population. 2015. https://www.ons.mr/images/RGPH2013/Chapitre01_R%C3%A9partition_spatiale_fr.pdf. Accessed 1 July 2020.

[28] Yacoub E, Tayfur G (2019). Trend analysis of temperature and precipitation in Trarza region of Mauritania. J Water Clim Change.

[29] Edwards FW (1941). Mosquitoes of the Ethiopian region. III. Culicine adults and pupae.

[30] Gillett JD (1972). The behaviour of mosquitoes and the transmission of human disease. Pestic Sci.

[31] Gillies M, Coetzee M (1987). A supplement to the Anophelinae of Africa south of the Sahara (Afrotropical region).

[32] Jupp PG (1996). Mosquitoes of Southern Africa: culicinae and toxorhynchitinae.

[33] Hervy JP, Le Goff G, Geoffroy B, Hervé JP, Manga L, Brunhes J (1998). The Anopheline mosquitoes of the Afrotropical region: an identification and training software.

[34] Tene Fossog B, Ayala D, Acevedo P, Kengne P, Abeso Mebuy IN, Makanga B (2014). Habitat segregation and ecological character displacement in cryptic African malaria mosquitoes. Evol Appl.

[35] Kengne P, Charmantier G, Blondeau-Bidet E, Costantini C, Ayala D (2019). Tolerance of disease-vector mosquitoes to brackish water and their osmoregulatory ability. Ecosphere.

[36] Morlais I, Ponçon N, Simard F, Cohuet A, Fontenille D (2004). Intraspecific nucleotide variation in *Anopheles gambiae*: new insights into the biology of malaria vectors. Am J Trop Med Hyg.

[37] Scott JA, Brogdon WG, Collins FH (1993). Identification of single specimens of the *Anopheles gambiae* complex by polymerase chain reaction. Am J Trop Med Hyg.

[38] Collins FH, Paskewitz SM (1996). A review of the use of ribosomal DNA (rDNA) to differentiate among cryptic *Anopheles* species. Insect Mol Biol.

[39] Kengne P, Awono-Ambene P, Antonio-Nkondjio C, Simard F, Fontenille D (2003). Molecular identification of the *Anopheles nili* group of African malaria vectors. Med Vet Entomol.

[40] Genetics Computer Group (GCG) (1997). Program manual for the GCG package, v. 9.1.

[41] Kumar S, Stecher G, Li M, Knyaz C, Tamura K (2018). MEGA X: molecular evolutionary genetics analysis across computing platforms. Mol Biol Evol.

[42] Senevet G, Andarelli L (1960). Contribution to the study of the biology of the mosquitoes in Algeria and in the Algerian Sahara. Arch Inst Pasteur Alger.

[43] Kirkpatrick TW (1925). The mosquitoes of Egypt: Egyptian government anti-malaria Commission.

[44] Senevet G, Andarelli L, Duzer A (1956). Breeding places of *Anopheles multicolor* in the region of Ténès (Algeria). Arch Inst Pasteur Alger.

[45] Mouchet J, Carnevale P, Coosemans M, Julvez J, Manguin S, Richard-Lenoble D (2004). Biodiversité du paludisme dans le monde.

[46] Trari B, Carnevale P (2011). De la préélimination à l’élimination du paludisme au Maroc. Quels risques pour l’avenir?. Bull Soc Pathol Exot.

[47] Guy Y. Les anophèles du Maroc. Rabat, Morocco: Société des Sciences Naturelles et Physiques du Maroc, Zoologie, Nouvelle Série No. 7; 1959.

[48] Le Sueur D, Sharp BL (1988). The breeding requirements of three members of the *Anopheles gambiae* Giles complex (Diptera: Culicidae) in the endemic malaria area of Natal, South Africa. Bull Entomol Res.

[49] Coluzzi M, Sabatini A (1969). Cytogenetic observations on the salt water species, *Anopheles merus* and *Anopheles melas* of the Gambiae complex. Parassitologia.

[50] Rejmánková E, Grieco J, Achee N, Roberts DR, Manguin S (2013). Ecology of larval habitats. Anopheles mosquitoes—new insights into malaria vectors.

[51] White BJ, Collins FH, Besansky NJ (2011). Evolution of *Anopheles gambiae* in relation to humans and malaria. Annu Rev Ecol Evol Syst.

[52] White BJ, Kundert PN, Turissini DA, Van Ekeris L, Linser PJ, Besansky NJ (2013). Dose and developmental responses of *Anopheles merus* larvae to salinity. J Exp Biol.

[53] Nwaefuna K, Bagshaw II, Gbogbo F, Osae M (2019). Oviposition and development of *Anopheles coluzzii* Coetzee and Wilkerson in salt water. Malar Res Treat.

[54] Jepson WF, Moutia A, Courtois C (1947). The malaria problem in Mauritius: the bionomics of Mauritian anophelines. Bull Entomol Res.

[55] Bradley TJ, Lancaster J, Briers RA (2008). Saline-water insects: ecology, physiology and evolution. Aquatic insects: challenges to populations.

[56] Coetzee M, Le Sueur D (1988). Effets of salinity on the larvae of some Afrotropical anopheline mosquitoes. Med Vet Entomol.

[57] Azrag RS, Babiker HM (2018). *Anopheles arabiensis* in Sudan: a noticeable tolerance to urban polluted larval habitats associated with resistance to temephos. Malar J.

